# Putative Epigenetic Biomarkers of Stress in Red Blood Cells of Chickens Reared Across Different Biomes

**DOI:** 10.3389/fgene.2020.508809

**Published:** 2020-11-02

**Authors:** Fábio Pértille, Adriana Mercia Guaratini Ibelli, Maj El Sharif, Mirele Daiana Poleti, Anna Sophie Fröhlich, Shiva Rezaei, Mônica Corrêa Ledur, Per Jensen, Carlos Guerrero-Bosagna, Luiz Lehmann Coutinho

**Affiliations:** ^1^Animal Biotechnology Laboratory, Animal Science and Pastures Department, University of São Paulo (USP)/“Luiz de Queiroz” College of Agriculture (ESALQ), Piracicaba, Brazil; ^2^Avian Behavioural Genomics and Physiology Group, IFM Biology, Linköping University, Linköping, Sweden; ^3^Embrapa Suínos e Aves, Concórdia, Brazil; ^4^Animal Science Program, Faculty of Animal Science and Food Engineering (FZEA), University of São Paulo (USP), Pirassununga, Brazil; ^5^Evolutionary Biology Centre, Department of Organismal Biology, Uppsala University, Uppsala, Sweden

**Keywords:** stress, red blood cells, epigenetics, chicken, DNA methylation, animal welfare

## Abstract

Production animals are constantly subjected to early adverse environmental conditions that influence the adult phenotype and produce epigenetic effects. CpG dinucleotide methylation in red blood cells (RBC) could be a useful epigenetic biomarker to identify animals subjected to chronic stress in the production environment. Here we compared a reduced fraction of the RBC methylome of chickens exposed to social isolation to non-exposed. These experiments were performed in two different locations: Brazil and Sweden. The aim was to identify stress-associated DNA methylation profiles in RBC across these populations, in spite of the variable conditions to which birds are exposed in each facility and their different lineages. Birds were increasingly exposed to a social isolation treatment, combined with food and water deprivation, at random periods of the day from weeks 1–4 after hatching. We then collected the RBC DNA from individuals and compared a reduced fraction of their methylome between the experimental groups using two bioinformatic approaches to identify differentially methylated regions (DMRs): one using fixed-size windows and another that preselected differential peaks with MACS2. Three levels of significance were used (*P* ≤ 0.05, *P* ≤ 0.005, and *P* ≤ 0.0005) to identify DMRs between experimental groups, which were then used for different analyses. With both of the approaches more DMRs reached the defined significance thresholds in BR individuals compared to SW. However, more DMRs had higher fold change values in SW compared to BR individuals. Interestingly, ChrZ was enriched above expectancy for the presence of DMRs. Additionally, when analyzing the locations of these DMRs in relation to the transcription starting site (TSS), we found three peaks with high DMR presence: 10 kb upstream, the TSS itself, and 20–40 kb downstream. Interestingly, these peaks had DMRs with a high presence (>50%) of specific transcription factor binding sites. Three overlapping DMRs were found between the BR and SW population using the most relaxed *p*-value (*P* ≤ 0.05). With the most stringent *p*-value (*P* ≤ 0.0005), we found 7 and 4 DMRs between treatments in the BR and SW populations, respectively. This study is the first approximation to identify epigenetic biomarkers of long-term exposure to stress in different lineages of production animals.

## Introduction

External influences affecting early life stages (pre- and post-birth) of animals can have enormous consequences on their adult phenotypes (Guerrero-Bosagna and Skinner, 2012). Several environmental factors are reported to affect early development, including environmental chemical compounds, endocrine disrupters (Susiarjo et al., 2013), inorganic chemicals (Kile et al., 2014), nutritional components (Dolinoy et al., 2007; Guerrero-Bosagna et al., 2008), or stress conditions (Fagiolini et al., 2009). Notably, epigenetic processes are substantially affected during early exposures to each of these factors (Guerrero-Bosagna and Skinner, 2012).

Epigenetics is the study of molecules that bind to DNA and can maintain this interaction in a mitotically stable manner (Skinner et al., 2010). Epigenetic modifications can regulate gene expression and are fundamental for all cellular processes. Epigenetic research has permeated several fields of biological research, from molecular to evolutionary biology (Richard and Stein, 2012). However, despite the importance of epigenetic mechanisms in biology in general, epigenetic studies in production animals are incipient, with some studies performed in chickens (Pértille et al., 2017a, 2019; Bélteky et al., 2018; Guerrero-Bosagna et al., 2020), in cattle (Su et al., 2014; Fang et al., 2017; Zhou et al., 2018; Sevane et al., 2019), in sheep (Cao et al., 2017; Capra et al., 2017; Zhang X. et al., 2017; Zhang Y. et al., 2017), and pigs (Gao et al., 2014; Choi et al., 2015; Yuan et al., 2017; Wang and Kadarmideen, 2019, 2020). Among production animals, chickens have recently emerged as a promising model for epigenetic studies (Frésard et al., 2013) following their historical use as a model for translational research with implications for human health and physiology (Kain et al., 2014).

Chickens are livestock animals of great zootechnical interest that are subjected to constant challenges in their production environment. These challenges include extreme temperatures, social disruption, unfamiliar sounds, unfamiliar or uncaring handlers, feed and water restriction injection with antigens, and diseases (Zulkifli, 2013). These exposures cause stress responses involving an initial homeostatic imbalance followed by physiological and behavioral responses (Zulkifli, 2013). Stress leads to immunodepression, reduced performance, and increased susceptibility to diseases (Eberhart and Washburn, 1993; Goerlich et al., 2012). Losses in poultry production are generated by some noticeable phenotypes such as carcass injury and dermatitis (Meluzzi et al., 2008) (which relate to poor rearing conditions) and to other phenotypes that are harder to notice, such as reduction in meat quality (Sandercock et al., 2001). Therefore, poor handling in production animals is an issue concerning not only animal welfare, but also meat quality and human nutrition. Consequently, it is of utmost importance to develop effective and cost-efficient tools to trace prolonged stress caused by poor practices in animal production.

By knowing the physiological and molecular mechanisms behind stress response in animals, it is possible to develop tools for its diagnosis. For example, under stress, alterations in the HPA axis activity result in elevated levels of glucocorticoids (Fallahsharoudi et al., 2015). Consequently, hormonal responses are produced such as changes in testosterone, noradrenaline, adrenaline, prolactin, adrenocorticotrophic hormone, and cortisol (Henry, 1993). Based on this plethora of hormonal changes, stress in animals is usually determined by hormone levels such as cortisol and epinephrine (Ishibashi et al., 2013; Müller et al., 2013). However, the release of stress hormones may show an acute physiological response but may not reflect a prolonged exposure to stress conditions (Henry, 1993). Recently, hair cortisol has been used as a long-term stress indicator (Heimbürge et al., 2019). In addition, salivary cortisol concentrations have also been used as a long-term stress indicator in horses (Peeters et al., 2011). Researchers have also begun to use corticosteroid metabolites in feathers, hair, or feces as a non-invasive metric of prolonged stress in different species (Berk et al., 2016). However, these authors report that procedures measuring hormones are laborious, the interpretation of the results is difficult, and the methods still need to be validated (Berk et al., 2016). Therefore, a major challenge still lies in determining the history of an organism’s exposure to stress for prolonged periods. Epigenetic marks in peripheral cells may serve as epigenetic biomarkers of stress history (Provencal et al., 2012; Wang et al., 2012) because in animals constantly subjected to stress it is expected that related hormonal plasmatic changes will leave an epigenomic mark in different cells, including blood cells.

One traceable epigenetic biomarker is the DNA methylation of cytosines in CpG dinucleotides (Feil and Fraga, 2012; Denham et al., 2016). We have recently reported DNA methylation differences in RBC of chickens reared in cages compared to conventional shed systems (Pértille et al., 2017a). However, in this proof-of-concept study we lacked the knowledge of which of these conditions was most stressful for the animals. The broad interest in RBC is due to the fact that they are easily purified and nucleated in birds. This allows us to easily extract DNA specifically from this cell type to study epigenetic changes without multiple cell bias and without producing major stress to the animal. In addition, other features of RBC have been investigated in relation to the physiological condition of organisms, including humans. For example, changes in RBC shape (Mueller et al., 2006) and distribution (Xanthopoulos et al., 2017) have been associated with several coronary, heart, and other systemic diseases in humans. Deformability of RBC is an essential feature of its biological function (Diez-Silva et al., 2010). Despite the importance of these studies in RBC, they are limited to the analysis of their phenotype. Studies in mammalian RBC involving DNA are very limited, since only immature RBCs are nucleated (O’Neill and Reddy, 2011). Therefore, the use of the chicken as a model is advantageous to understanding possible biological mechanisms involving erythrocyte responses in vertebrates.

A robust epigenetic biomarker of stress should be reproducible between animals raised in different environmental conditions (biomes). Thus, the present study is based on three strategies in order to validate and fill the gaps opened by our previous study (Pértille et al., 2017a). The present study involves the exposure of chickens from a genetically controlled population to a known stress. First, we selected one of the most utilized chicken breed lines around the world, i.e., White Leghorn. White Leghorn is a Mediterranean breed widely used throughout the global egg industry. All commercial white egg lineages have originated from line crosses involving the White Leghorn breed (Fulton, 2006). Second, we selected lineages with similar genetic backgrounds and used the same protocol to induce a known common stressor (Yanagita et al., 2011; Goerlich et al., 2012) that involves social isolation and deprivation of food and water stress. Third, the animals were reared in completely different biomes, namely in experimental systems in Sweden and a validation population in Brazil. We compared DNA methylation in a reduced fraction of the RBC genome (obtained by genomic digestion using a restriction enzyme) between individuals in a stress and a non-stress condition and then we compared the effects across the two different biomes.

The results of the present study have an important impact from the point of view of animal welfare. It brings us closer to the identification of epigenetic markers that would test the history of stress that production animals experience in their housing environment.

## Materials and Methods

All animal experimental protocols employed in the present study were performed in accordance with international guidelines for animal welfare. In Brazil, the study was approved by the resolution #008/2017 from the CEUA following the CONCEA. In Sweden the study was approved under the license #50-13 from the Regional Committee for Scientific Research on Animals from Linköping, Sweden.

### Animal Rearing and Treatments

This study was conducted using non-beak-trimmed, male White Leghorn chickens (*Gallus gallus domesticus*) aged 0–26 days with normal health status. The experiments were performed with chickens from, and in, different geographic locations. One of the lineages was from Brazil (BR) and the experiment was performed at the Embrapa Swine and Poultry National Research Center, Concórdia/Santa Catarina State. The other population was from Sweden (SW) and the experiment was performed at Linköping University. In Brazil, birds were hatched at the EMBRAPA experimental hatchery, while in Sweden, birds were hatched at a commercial hatchery and immediately brought to a rearing farm. In each location, all birds were housed within the same room, where they were provided with *ad libitum* access to water and to feed provided through tubular feeders in BR, and a chain dispersal system in SW. The commercial diet provided for the laying birds in BR contained 12.1 MJ/kg ME (metabolizable energy) and 21.41% CP (crude protein), while the diet provided to the birds in SW contained 11.3 MJ/kg ME and 15.5% CP. The animals were raised in conventional sheds in BR while in a dark house in SW. Thus, the experimental settings present many differences related to the different biomes (different weather, rearing systems, and diets for each country) and minimize the experimental heterogeneity within the same biome.

A social isolation treatment (S) was performed on 8 out of 14 animals in the SW experiment (6 controls) and on 16 out of 32 animals (16 controls) in the BR experiment.

This treatment consisted in exposing birds of both SW and BR populations to social isolation stress at random times once a day between 4 and 26 days of age. During the treatment, these birds were individually placed in a metal (25 cm × 38 cm × 18 cm) mesh (in SW facilities) or wooden (28 cm × 28 cm × 30 cm) boxes (in the BR facilities). There, they had vocal, but not visual or physical contact with other birds. Thus, during the stress treatment the birds were actually exposed to a combination of stressors: social isolation and deprivation of food and water. The time in the treated box was gradually increased from 1 h during the first week to 2 h during the second and 3 h during the third week. The control animals (C) were not exposed to the sessions of social isolation stress but experienced the same environmental conditions as S animals. Blood samples from the S and C animals were collected 2 h after the last isolation. The same experimental set up has been previously applied (Goerlich et al., 2012) to measure the effects on corticosterone levels and gene expression across generations. Thus, the stress effect induced by this experimental design has already been validated. To account for genetic effects in the BR experiment, we randomly separated complete siblings between the S and C groups, i.e., for each chicken randomly chosen for the S group, one random sibling was chosen for the C group.

### Collection of Biological Material

Two mL of blood were collected in a 2 mL microtube with 20 μL of 0.5 M EDTA from each individual. After blood collection, each tube was centrifuged at 3,000 rpm for 5 min and the RBC fraction was separated from the whole blood. The upper white and yellow fractions were discarded. After this, the blood samples were frozen at −20°C.

The DNA from the RBC was extracted after digestion with proteinase K (Promega; Madison, United States), precipitation of DNA in absolute ethanol, washing of DNA in 70% ethanol, and re-suspension in ultrapure water. The DNA samples were quantified in a fluorometer (Qubit Fluorometric Quantitation). The DNA quality was evaluated using the Nanodrop 2000c spectrophotometer and the integrity was checked on 1% agarose gels.

### Preparation of Sequencing Libraries

To prepare the sequencing libraries, we used an approach that combines two techniques previously optimized in chickens: GBS (Pértille et al., 2016) and MeDIP (Guerrero-Bosagna and Jensen, 2015). We recently described the optimization of this methodological combination in previous studies (Pértille et al., 2016, 2017a). This approach allows for the parallel identification of genetic and epigenetic differences between experimental groups in the same reduced fraction of the genome across individuals. The general idea of this method is to assess genome wide levels of methylation in a reduced fraction of the genome that is not biased toward CpG islands, using a restriction enzyme unrelated to CpG sites. With our method, we first digest the genome with the *PstI* restriction enzyme (Thermo Scientific; Waltham, MA, United States) in a suitable range (∼450 bp) for Illumina (San Diego, CA, United States) sequencing (Pértille et al., 2016). Illumina sequencing barcodes are then ligated to each end of the digested DNA fragments, allowing the pool of DNA samples to be immunoprecipitated together. Each pooled DNA sample contained different barcodes identifying each individual. The methylated fraction of the sampled DNA is then captured by an anti-methyl-cytosine antibody (Diagenode; Sparta, United States) (Guerrero-Bosagna and Jensen, 2015). After this step, the methylated DNA is amplified using PCR, which is followed by a clean-up of the primer dimers and unbound adapters (Elshire et al., 2011; Poland et al., 2012). The samples are then sent for sequencing. A detailed description of the protocol related to this combination of methods is currently being prepared for publication elsewhere. In BR samples, after connecting the DNA to adapters and barcodes, and pooling the samples, a 50 ng fraction of the DNA pool was taken for immediate amplification by PCR. This portion represents the genetic background of the libraries, which we call inputs. Moreover, after library prep, for the BR samples, each library was quantified by quantitative PCR using the KAPA Library Quantification Kit (Roche; Basel, Switzerland). Sequencing libraries were diluted to 16 pM and clustered using the cBOT (Illumina; San Diego, CA, United States) equipment. Paired-end sequencing with a read length of 100 bp was performed using the HiSeq2500 instrument from Illumina in the BR lab facility. In SW samples, we sent out the libraries to be quantified, clustered, and paired-end sequenced in the Illumina HiSeq2500 platform with a read length of 125 bp at the SNP&SEQ facilities of the SciLifeLab (Uppsala, SE).

### Bioinformatic Analyses

The CASAVA (Illumina) program was used for the initial processing of the samples by converting the “.bcl” (base call files) to “.fastq” extensions, which is compatible with programs used for read alignment. The quality of the reads was checked using FastQC v.0.11.3 (Andrews, 2010). Quality trimming was performed in short read sequences during the data processing using default parameters. For both SNP calling and methylation analyses, quality-trimmed reads were aligned against the chicken reference genome (*Gallus_gallus* 5.0, NCBI) using the Bowtie2 tool v.2-2.3.4.2 (Langmead and Salzberg, 2012) default parameters. The coverage depth of each sequenced file was determined using Samtools v.0.1.19 (Li et al., 2009) with the “depth” option. A TASSEL-GBS Discovery Pipeline was used to process the data and for SNP calling by employing default Tassel v.3.0 (Glaubitz et al., 2014) parameters. For SNP calling, default filtering parameters were applied except for the use of 5% for mnMAF, 70% of mnTCov, and 70% for mnScov. These parameters were applied in accordance with similar studies (Pértille et al., 2016, 2017a, b). An Archaeopteryx tree (Han and Zmasek, 2009) was then plotted using a cladogram generated by a Neighbor Joining distance matrix using the Tassel v.3.0 software. For the identification of differential methylation regions (DMR), uncalled and low quality score bases were eliminated using the process_radtags function in the Stacks v.1.39 (Catchen et al., 2011) program.

After the alignment, the .bam files from each individual were then assigned to one of the experimental groups (S or C). DMRs were then calculated between the two experimental conditions using the MeDIPS package within R (Lienhard et al., 2014) for basic data processing, quality controls, normalization, identification of differential methylated regions (DMRs), and the calculation of fold-changes methylation values among experimental groups. We used MeDIPS with default parameters and the BSgenome.Ggallus.UCSC.galGal5 (package from Bioconductor). In addition, to identify confounding factors related to the input (i.e., copy number variations, CNVs), we tested for differential coverage between the inputs of S and C samples in BR individuals. MeDIPs allows us to include the following parameters: two methylation enriched DNA sets (named MSet S and C) and two input sets (named ISet S and C). When the two methylation-enriched DNA sets are included, MeDIPs calculates DMRs. In addition, when two input sets are included, MeDIPs will identify CNVs between the inputs and match the results to the DMRs identified between the two methylation-enriched sets^1^. This gives an output containing only the DMRs that do not match with the CNVs calculated from the inputs.

Quality control was carried out to confirm the enrichment of the methylated fraction of the genome. This was performed by calculating the average enrichment score. Enrichment scores > 1 are recommended in the literature to denote methylated DNA enrichment, with values around 2 being optimal (Lienhard et al., 2014).

**FIGURE 1:**
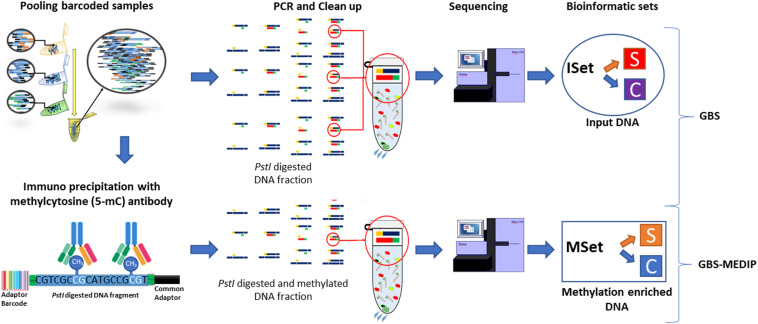
Scheme depicting the general experimental workflow of the genetic and methylomic analyses employed and the datasets used for the bioinformatic analyses.

We followed the same specific parameters for the MeDIPS package previously used (Pértille et al., 2017a). However, to call DMRs we used two different approaches in which the parameter *P* = 0.01 was used as the threshold for the detection of stacked reads. The first approach was the MeDIPS default in which the genome is divided into ADJW of a pre-defined length size of 100 bp. This differential methylation analysis then uses a weighted trimmed mean of the log expression ratios (TMM) (Robinson and Oshlack, 2010). In the second approach, a pre-selection of the regions later tested in MeDIPS was obtained by comparing the methylation enriched sets (Msets S and C) using the MACS2 peak calling program^2^ (Zhang et al., 2008) with default parameters. In MeDIPs, differences between the methylation enriched sets (Msets), or between the input sets (Isets), were tested only on these MACS2-generated regions. MACS2 is a recommended tool to identify sample-wise ‘peak specific’ methylated regions of variable sizes in experiments using paired controls to determine enrichment against background (Feng et al., 2012; Niazi et al., 2016; Cavalcante et al., 2019). The use of MACS2 contrasts to the division of the genome into small subsequent windows performed by the ADJW approach. Importantly, these peaks, called ‘regions of interest’ (ROI), were obtained after passing FDR correction (≤0.1). After ROIs were identified, MeDIPs used them to test for DMR. ROIs found differentially methylated by MeDIPS were called ROI-DMRs (*P* ≤ 0.05). MACS2 improves the spatial resolution of the predicted sites, uses a dynamic parameter to capture local biases in the genome, and improves the robustness and specificity of the prediction, that are strongly indicated for fold-enrichment experiments (Zhang et al., 2008). Considering that in the BR individuals the sequencing covered a ∼3.5 times larger region than the sequencing of SW individuals, we performed differential weighting of default parameter thresholds for a minimum sum of counts across all samples per window. Therefore, for SW individuals we used the default parameter (minRowSum = 10) and for BR individuals we used a 3.5 times higher value (minRowSum = 35). As a consequence of this differential weighting, a similar number of windows between individuals from Brazil and Sweden were tested using both the ADJW and ROI approaches. For a general visualization of the experimental and bioinformatic workflow see Figure 1, and Supplementary Figure 1.

The differential genomic windows obtained by the ADJW and ROI analyses were considered as DMRs based on three significance thresholds: *P* ≤ 0.0005 DMRs were used for describing genes related to significant DMRs; *P* ≤ 0.005 DMRs were used for visualization purposes and to describe overlapping DMRs between the ROI and ADJW approaches; and *P* ≤ 0.05 DMRs were used for enrichment pathway analyses, for the detection of overlapping DMRs between the BR and SW lineages, and to calculate distance among neighboring DMRs and between DMRs and their nearest TSS. ROIs that passed a significance threshold level of 0.05 were considered ROI-DMRs. The idea behind using datasets with different levels of significance was to obtain different levels of information. For example, more stringent *p*-values will give a better idea of specific sites to be tested in the future as putative epigenetic biomarkers of stress in RBCs. Less stringent *p*-values will give information of which biological pathways could be influenced by the early stress applied, and also provide DMRs to be tested later as common epigenetic regions altered by the stress in different populations. All DMRs obtained were annotated against the chicken reference genome (*Gallus_gallus* 5.0, NCBI) using the VEP tool (McLaren et al., 2010).

In addition to the VEP annotation, we performed an annotation with R in order to obtain information about the distance between each ROI-DMR and their nearest TSS. For this, we extracted the coordinates from each ROI (from the BR and SW populations) and from the annotated genes from the chicken genome (using the org.Gg.eg.db package). Then, we overlapped the identified ROI with these annotated genes using the Genomic Ranges R package. Next, we performed a functional genomic annotation of the ROIs overlapping with genes. For this, we used the annotatePeak function from the ChIPseeker package (Yu et al., 2015). In this function, we forged a gg_txdb object using the GenomicFeatures and org.Gg.eg.db packages. The latter is the functional annotation database for the chicken genome (BSgenome.Ggallus.UCSC.galGal5). This gg_txdb for *Gallus gallus* was extracted from the transcript metadata TxDB, which contains all the functional annotations available at the UCSC genome browser. For this, we used the function makeTxDBFromUCSC (using the parameter: genome = “galGal5”).

After identifying specific features related to DMRs found in each population (BR and SW) we checked the distribution of the identified ROI-DMRs in relation to (i) the nearest ROI-DMR, and (ii) the nearest gene’s TSS locations. For this, we considered different chromosomal data subsets: all, autosomal, and Z chromosomes. These distances were categorized in ten, hundred, thousand, and million numerical magnitudes. The analysis includes every distance that was counted at least once. At continuation, we tested whether transcription factor binding site (TFBS) motifs could map to the ROI-DMRs associated to specific peaks of distance in relation to the TSS. This test was performed with the web-based tool PhysBinder^3^ (Broos et al., 2013) using the threshold of ‘Max. Precision (PPV)’ and selecting all the 85 transcription factors available^4^, which included all ‘Direct Evidence’ and ‘Putative Associated’ factors. The motifs selected as of special interest were those that passed two criteria: TFBS should have hits in all DMRs longer than 100 bp tested (even if non-significant) and should have hits above the threshold (i.e., considered significant) in at least 50% of the DMRs tested.

An overlap analysis to identify DMRs obtained by both methods, i.e., ADJW and ROI (in the two different experiments) was performed by permutation tests (*N* = 100) that determined which peak overlaps were significant. For this, we used the findOverlapsOfPeaks function from the ChIPpeakAnno v3.6.5 R package with default parameters. Venn diagrams were plotted using the *makeVennDiagram* function within the same package.

The internet-based tool used to identify over-represented pathways related to our gene list was Reactome v72 (Croft et al., 2011). This is an open-source curated bioinformatic database of pathways and reactions from human and other animals^5^. Reactome is capable of accessing a variety of databases that contain previously described biological pathways (e.g., Kegg, Biocarta, Reactome, and Wikipathways).

## Results

### Sequencing and Alignment

The average sequencing and alignment statistics for the individuals investigated in the SW (*N* = 14) and BR (*N* = 32) experiments are shown in Table 1. More details can be found in Supplementary Tables 1, 2. For BR White Leghorn chickens, we sequenced both the reduced genome (*N* = 32 and 16/treatment) and its methylated fraction (*N* = 32 and 16/treatment). The reduced genome, obtained through the GBS approach, was used as the input for genetic background analyses. The individually reduced genomes were used to verify the relatedness among individuals. For SW White Leghorn chickens (*N* = 14 and 8/treatment) we did not use input DNA, because the idea with this population was only to compare their RBC methylomic response (to social isolation) with BR chickens.

**TABLE 1:** Average sequencing and alignment statistics for the White Leghorn SW and BR lineages.

Lineages	Samples	Sample number	Depth ± *SD*	Number of bp sequenced ± *SD*	Breadth (bp sequenced/depth) ± *SD*	% of the *Gal.gal* genome v.5.0 covered ± *SD*
SW	MeDIP average	14	54.7 ± 5.5	313,562,292.1 ± 112,051,857.5	5,786,017.2 ± 2,201,546.4	0.54 ± 0.20
BR	MeDIP average	32	30.2 ± 3.1	597,344,785.8 ± 294,204,155.2	20,026,886.1 ± 10,085,067.7	1.9 ± 0.9
	Input average	32	23.0 ± 3.4	741,551,215 ± 186,608,136	31,947,116.0 ± 3,230,589.0	2.97 ± 0.30

### DNA Background

**FIGURE 2:**
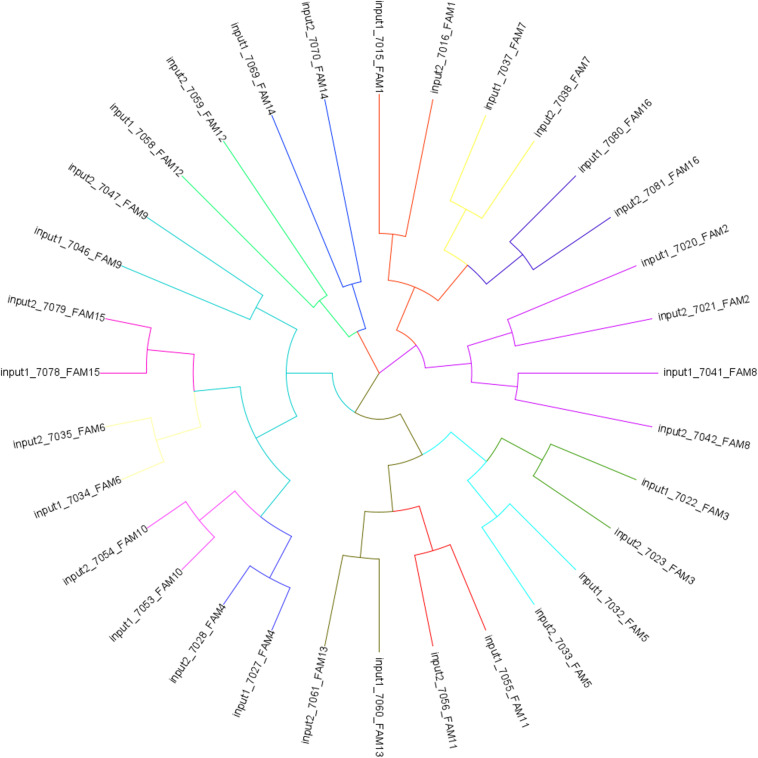
Cladogram tree generated by a Neighbor Joining distance matrix from SNPs identified across individuals from input 1 (group C) and 2 (group S).

Based on the input DNA of all BR individuals, we identified 93,215 SNPs among them (Supplementary VFC File 1 online). From these SNPs, a tree showing genetic relatedness was plotted using a cladogram generated by a Neighbor Joining distance matrix (Figure 2). The cladogram in Figure 2 shows that individuals are grouped by family, which is expected because we controlled the genetic background by separating the full siblings between S and C groups. Each individual analyzed was categorized within the expected family cluster (tree branch) according to their genetic relationship. The .bam files generated by the genomic sequences of the individuals were later used for methylomic coverage normalizations.

### CpG Enrichment

For BR individuals, an enrichment score of 1.59 (±0.13) was obtained and, in average, 3.94% of the CpG regions on the *Gallus gallus* genome were covered (Supplementary Table 3). For SW individuals we obtained an enrichment score of 2.87 (±0.10) and, in average, 1.18% of the CpG regions on *Gallus gallus* genome were covered (Supplementary Table 4). This indicates that in BR genomes approximately 3.33 × (3.94/1.18) more CpG regions were covered than in SW genomes. This difference could be explained by a different procedure conducted in the SW library construction compared to the BR library. In SW libraries, the protein beads containing immunoprecipitated DNA were washed once more than what was previously recommended (Guerrero-Bosagna and Jensen, 2015). Concomitant with the fact that the number of covered regions were lower in SW samples, coverage depth was higher. Consequently, a reduction in false positive and an increase in false negative DMRs is expected to be observed in the SW population.

### Analysis of Differentially Methylated Regions (DMRs)

Three levels of significance were used to identify DMRs between the S and C groups (*P* ≤ 0.05, *P* ≤ 0.005, and *P* ≤ 0.0005). These were then used for different analyses. The number of DMRs obtained with each significance level used is shown in Table 2. Windows obtained with the less stringent values of *P* ≤ 0.005 and *P* ≤ 0.05 were used to investigate overlaps among the different methodologies employed, i.e., ROI and ADJW (Supplementary Spreadsheet 1). Additionally, DMRs obtained with *P* ≤ 0.05 were selected to identify common DMRs between the two different experiments, since no overlap was obtained with more stringent *p*-values. Although *P* ≤ 0.05 will generate more false positives, it can also provide a set of putative regions to be further investigated with other methodologies. Windows obtained with *P* ≤ 0.0005 when using the ADJW approach were considered of special relevance in terms of significance and the ADJWs fulfilling this criterion were merged (Table 3).

**TABLE 2:** Total number of genomic windows sequenced and tested in the BR and SW lineages and number of DMRs identified using different *p*-value thresholds.

Test		BR	SW	BR	SW	BR	SW	BR	SW
						
minRowSum		35	10	*P* ≤ 0.0005		*P* ≤ 0.005		*P* ≤ 0.05	
ADJW (100 bps windows)	Total	10,188,209	10,188,209	6	7	38	57	359	561
	Tested	14,040	14,522						
ROI (FDR ≤ 0.1)	Total	73,825	12,019	4	1	17	6	154	91
	Tested	5,058	994						

*Genomic windows were tested when they passed a “minimum sum read count” parameter of 10 for SW and of 35 for BR experiments.*

**TABLE 3:** Putative stress-induced DMR descriptive statistics and gene annotations for the DMRs found with the ADJW and ROI (*P* ≤ 0.0005) analyses in BR and SW populations (SvsC groups).

S vs. C	Test	Location	Width	logFC	*P*-value	FDR *p*-value	CpGs	% of CpG	Position regarding gene	Gene symbol	ENSEMBL name	Gene strand
BR	ADJW	chr1:61077901-61078200	300	3.4	2.24E-05	1.05E-01	19	6%	Intron/intron	*LRTM2*/*NA*	ENSGALG00000026214/	1/−1
											ENSGALG00000036964	
		chr6:17299601-17299700	100	2.1	4.77E-04	1.00E+00	1	1%	Intergenic	–	–	–
		*chr28:3263201-3263400	200	3.5	2.33E-06	1.64E-02	7	7%	Upstream gene/downstream gene	*ADAMTSL5*/THOP1	ENSGALG00000024298/	−1/−1
											ENSGALG00000041934	
	ROI	chr9:3693375-3694655	1281	1.4	4.76E-04	5.84E-01	44	3%	Coding sequence, intron	*KY*	ENSGALG00000044975	−1
		chr26:3262317-3263255	939	1.6	5.91E-05	1.49E-01	30	3%	Downstream gene	–	ENSGALG00000001480	−1
		*chr28:3263299-3263412	114	3.4	3.40E-06	1.72E-02	9	8%	Upstream gene/downstream gene	*ADAMTSL5*/THOP1	ENSGALG00000024298/	1
		chrZ:7337093-7337617	525	1.5	4.76E-04	5.84E-01	6	1%	Intron	*UBE2R2*	ENSGALG00000041934	1
SW	ADJW	chr14:12337401-12337500	100	3.2	1.93E-04	4.00E-01	3	3%	Downstream gene/downstream gene	*CLCN7*/*NA*	ENSGALG00000030826/ENSGALG00000032279	−1/1
		chr20:49301-49600	300	−4.4	4.18E-05	2.03E-01	24	8%	Intergenic	–	–	–
		chrZ:70400401-70400700	300	−.5.4	1.46E-04	3.53E-01	2	1%	Intergenic	–	–	–
	ROI	chr2:66704195-66704415	221	5.5	3.56E-04	3.53E-01	4	2%	Intron	*IRF4*	ENSGALG00000012830	1

*overlaps among methodologies (ROI and ADJW); “/” separate genes that are associated to the same DMR; NA means information not available. DMRs shown for ADJW represent merged consecutive windows significantly altered.

With ADJW we investigated relative DNA methylation changes between experimental groups in 10,188,209 ADJW of 100 bps across the chicken genome. A total of 359 and 561 of these windows were obtained with a threshold of *P* ≤ 0.0005 for BR and SW experiments, respectively (Table 3). Merging of these adjacent differentially covered windows identified by ADJW between the S and C groups produced 3 DMRs in the SW experiment and 3 DMRs in the BR experiment. Of all DMRs identified by ADJW one passed FDR ≤ 0.05 (FDR = 0.02), which was identified in the BR population (Table 3).

In parallel, with MACS2 we identified 14,040 regions with differential coverage in the methylation enriched genomes between S and C groups in the BR experiment, and 14,533 in the SW experiment (Table 2). After testing these regions with the MeDIPS package, 4 and 1 ROI-DMRs were obtained with a threshold of *P* ≤ 0.0005 for the BR and SW experiments, respectively (Table 3). Of all ROI-DMRs one passed FDR ≤ 0.05 (FDR = 0.0000034), which was identified in the BR population (Table 3). Importantly, this is the same region that was previously identified to pass FDR ≤ 0.05 with ADJW.

**FIGURE 3:**
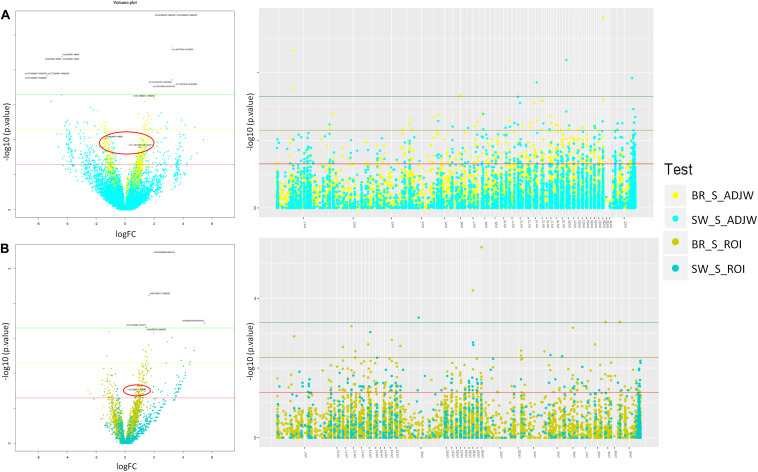
Volcano and Manhattan plots showing stress-induced DMRs found in BR and SW lineages using **(A)** ADJW and **(B)** ROI. Thresholds shown correspond to *p*-values 0.05 (red), 0.005 (yellow), and 0.0005 (green). Overlapping windows in Volcano plots are marked in red. Light colors represents ADJW and dark colors represent ROI.

**FIGURE 4:**
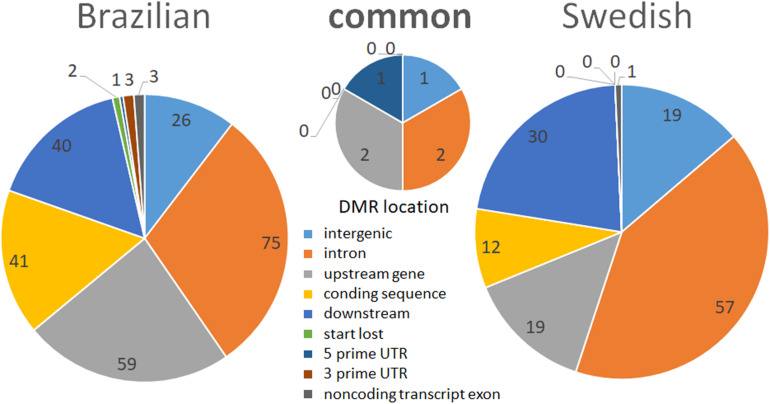
Pie charts representing the functional annotation of stress-induced ROI-DMRs in relation to genes in the reference chicken genome. The left pie represents ROI-DMRs identified in the BR experiment and the right pie represents ROI-DMRs found in the SW experiment. The middle pie chart shows the ROI-DMRs overlapping between lineages.

Additionally, in the BR population we tested for artifacts potentially caused by differential coverage of the input between the S and C groups using the inputs as the Iset parameter in the MeDIPs package (Lienhard et al., 2014). These coverage differences could mask or enhance DMR identification. The results were unchanged after using the inputs to control for eventual pre-existing coverage differences. In addition to the ROI-DMRs obtained between the S and C groups with a threshold of *P* ≤ 0.0005 (1 in SW and 4 in BR), using a less stringent *p*-value (*P* ≤ 0.05) we identified 359 ROI-DMRs in the BR experiment and 561 ROI-DMRs in the SW experiment. About 50% of these ROI-DMRs overlapped with DMRs identified with AJDWs (*P* ≤ 0.05). Detailed information of the DMRs above the two thresholds depicted in Figure 3 are shown in Tables 3, 4. All ROI-DMRs were annotated using the VEP tool. Additionally, “consequences” were plotted using pie charts for a general view of their location in relation to the annotated genes in the chicken genome (including those overlapping test) (Figure 4).

**TABLE 4:** Descriptive statistics and gene annotations for ROI DMRs found (*P* ≤ 0.005) in BR and SW populations (S vs. C groups).

S vs. C	ROI ∼ DMR	Location	Width	LogFC	*P*-value	FDR p-value	CpGs	% of CpG	Position regarding gene	Gene symbol	ENSEMBL name	Gene strand
BR	T	chr1:61077945-61078198	254	2.9	1.23E-03	8.91E-01	16	6%	Intron/intron	*LRTM2*/NA	ENSGALG00000026214/	1/−1
											ENSGALG00000036964	
	T	chr2:189007-189224	218	1.4	2.32E-03	1.00E+00	11	5%	Downstream gene	–	ENSGALG00000043576	−1
	T	chr4:740034-740180	147	1.5	4.36E-03	1.00E+00	1	1%	Intergenic	–	–	
	F	chr4:1212081-1212352	272	−1.3	3.15E-03	1.00E+00	6	2%	coding sequence/3 prime UTR	–	ENSGALG00000004475	1
									Upstream gene/upstream gene	*WNT11B/gga-mir-6658*	ENSGALG00000004401/ENSGALG00000028208	1/−1
	T	chr4:85246930-85247063	134	1.5	3.36E-03	1.00E+00	0	0%	Intron	*CTBP1*	ENSGALG00000039942	1
	T	chr6:17299560-17299780	221	2	6.93E-04	5.84E-01	4	2%	Intergenic	–	–	–
	F	chr8:5191540-5191738	199	1.8	2.10E-03	1.00E+00	18	9%	Coding sequence, intron	*LMX1A*	ENSGALG00000003424	1
	F	chr9:3693375-3694655	1281	1.4	4.76E-04	5.84E-01	44	3%	Coding sequence, intron	*KY*	ENSGALG00000044975	−1
	F	chr11:1715302-1715998	697	1.5	2.47E-03	1.00E+00	9	1%	Coding sequence, intron	*VAC14*	ENSGALG00000002458	1
	F	chr12:2700294-2700543	250	−1.1	6.30E-04	5.84E-01	11	4%	Coding sequence, intron	–	ENSGALG00000046133	1
									Downstream gene	–	ENSGALG00000046133	1
	F	chr12:16285799-16286230	432	1.4	4.99E-03	1.00E+00	7	2%	Intron, non -coding transcript	–	ENSGALG00000035037	1
	T	chr12:16763013-16763444	432	1.4	3.27E-03	1.00E+00	16	4%	Upstream gene	*PDZRN3*	ENSGALG00000007819	−1
	T	chr14:4134485-4134909	425	1.3	2.09E-03	1.00E+00	45	11%	Start lost, start retained, 5 prime UTR	*TNRC18*	ENSGALG00000040770	−1
	F	chr18:10291116-10291837	722	1.6	1.54E-03	9.71E-01	41	6%	Coding sequence, intron	*CACNA1G*	ENSGALG00000007623	−1
	F	chr26:3262317-3263255	939	1.6	5.91E-05	1.49E-01	30	3%	Downstream gene	*–*	ENSGALG00000001480	−1
	T	chr28:3263299-3263412	114	3.4	3.40E-06	1.72E-02	9	8%	Upstream gene/downstream gene	*ADAMTSL5/THOP1*	ENSGALG00000024298/	1
											ENSGALG00000041934	
	F	chrZ:7337093-7337617	525	1.5	4.76E-04	5.84E-01	6	1%	Intron	*UBE2R2*	ENSGALG00000001668	1
SW	F	chr2:66704195-66704415	221	5.5	3.56E-04	3.53E-01	4	2%	Intron	*IRF4*	ENSGALG00000012830	1
	T	chr5:5717497-5717944	448	3.9	4.23E-03	4.79E-01	9	2%	Intergenic	*–*	–	
	F	chr5:48281849-48282123	275	4.5	4.61E-03	4.79E-01	16	6%	Intergenic	*–*	–	
	T	chr14:12337347-12337639	293	3.2	9.29E-04	4.62E-01	10	3%	Downstream gene/downstream gene	*CLCN7/NA*	ENSGALG00000030826/ENSGALG00000032279	−1
	T	chr26:3554060-3554373	314	4.2	1.85E-03	4.79E-01	15	5%	intron	*RHOC*	ENSGALG00000001569	−1
									Downstream gene/downstream gene	*MOV10/PPM1J*	ENSGALG00000001558/ENSGALG00000001605	1
	T	chr26:4552858-4553138	281	4.8	2.18E-03	4.79E-01	0	0%	Downstream gene	*OPN1MSW*	ENSGALG00000002848	–

*T (true) and F (false) when the ROI is overlapped or not, respectively, by an AJDW identified using the same P-value threshold; “/” separate genes that are associated to the same DMR; NA means information not available.*

With both of these approaches more DMRs reached the defined significance thresholds in BR individuals compared to SW. However, more DMRs had higher fold change values in SW compared to BR individuals. When comparing the two approaches used to call DMRs (Figure 3), we observed that the number of DMRs identified with ADJW was 4 times larger than with ROI (Table 2). This was probably due to the fragment size analyzed, since with ADJW a fixed 100 bp window was used, while with ROI the average fragment sizes in BR and SW groups were 323 ± 256 and 427 ± 479 bps, respectively (according to the peak calling). Expectedly, ROI-DMRs tend to encompass larger regions than ADJW-DMRs (fixed in 100 bps). Not surprisingly, when using the ADJW method some DMRs were identified that had no CpGs. The reason for this is that DMRs larger than 100 bp identified by ADJW will be arbitrary divided into 100 bp sub windows and not all of these sub-windows will have a CpG in their composition. Due to this, the ADJWs were merged (see Table 3). Additionally, CpG count can equal zero when the CpG is present in the analyzed population but not in the reference genome (see Table 4). Another difference is that ROI tended to detect more hypermethylated DMRs in the S vs. C groups than ADJW. With a stringent *p*-value threshold (*p* ≤ 0.0005) no overlaps were found. With a less stringent threshold of *P* ≤ 0.005, 47.1% of the DMRs identified by ROI overlapped with DMRs identified by ADJW in the BR experiment, while 62.3% of the ROI and ADJW DMRs identified overlapped in the SW experiment. Considering *P* ≤ 0.05, this overlap corresponds to 66.7 in BR, and 49.5%, in SW.

**FIGURE 5:**
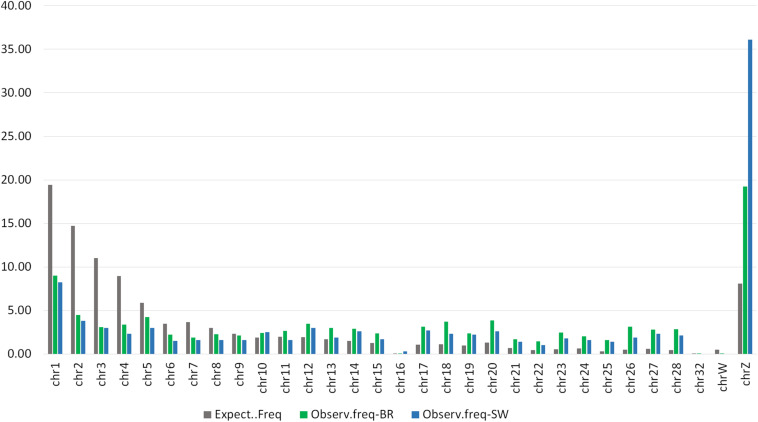
Distribution expectancy of random 100,000 windows of 237 ± 184 bps across the *Gallus gallus* chromosomes compared with the observed distribution of 5,058 and 994 peaks called between the S and C groups of the BR and SW populations, respectively (ROI).

To verify the distribution of the DMRs throughout the chicken genome we first constructed a Manhattan plot (right side of Figure 3) with the three above-mentioned thresholds of significance. Additionally, we plotted a distribution expectancy of 100,000 random windows across the chicken genome to compare with the observed distribution of the 5,058 and 994 peaks called between S and C groups in BR and SW (ROI peaks) populations, respectively. For this, we used 237.7 ± 184 bps mean length, which was the average length of all windows used in our study (ROI peaks for BR and SW). An inverse distribution of the observed DMRs was identified in relation to their random expectation corrected by chromosome size (Figure 5). This distribution of DMRs increases toward microchromosomes. We analyzed the sex ChrZ separately because even though it is considered a macrochromosome in chicken (Habermann et al., 2001), it presented the greater proportion of DMRs. Interestingly, the ChrZ was enriched to 2.38× (BR) and 4.47× (SW) above expectancy for the presence of DMRs (Figure 5). Additionally, to eliminate any remaining possibility of this being an artifact of our technique, we assessed the number of expected CpGs and *PstI*-digested fragments (ranging from 200 to 600 bps – compatible with our library size) per chromosome in relation to their size. Neither the number of CpGs nor the number of *Pst*I-generated fragments was statistically different than expected (*t*-test *P* ≤ 0.05).

In relation to the distances among ROI-DMRs, we found that most distances were concentrated at 1–2 Kbps, 20–30 Kbps, 200–300 Kbps, and 10 Mbps in both populations (Supplementary Spreadsheet 2 online). Altogether, these peaks represent 52.9% (BR) and 57.9% (SW) of all ROI-DMRs within the respective population.

**FIGURE 6:**
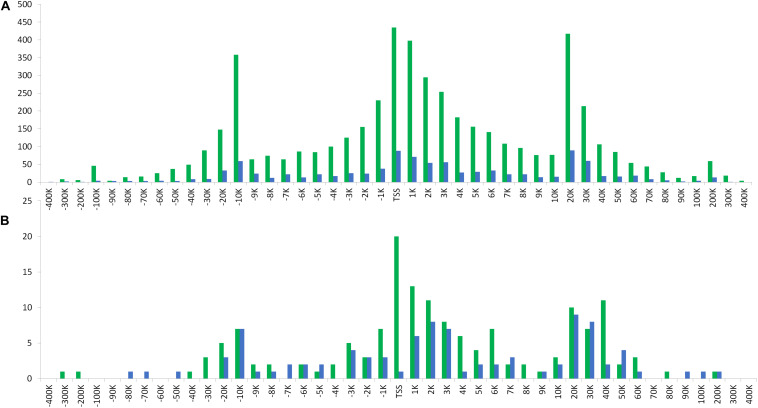
Distribution of **(A)** the ROI peaks and, **(B)** the ROI-DMRs for each analyzed population (BR and SW) in relation to the nearest TSS location.

In relation to the distribution of distances from ROI-DMRs to their nearest TSS, we observed high similarity between the two populations (BR and SW) studied (*r* ≥ 0.94). The same trend was observed between the chromosome subsets analyzed (all vs. autosomal and all vs. ChrZ) within each population studied (*r* ≥ 0.93). Both distributions of distances between either ROI or ROI-DMRs and their nearest TSS presented a trimodal pattern (Figure 6 and Supplementary Spreadsheet 2 online). The highest peaks of these trimodal distributions were located at −10 Kbps, 0 Kbps, and +20 Kbps from the TSS. The exception was the largest distance peak between ROI-DMRs and TSS in the SW population, located at 40 Kbps.

The observation of these peaks of ROI-DMRs in relation to the TSS together with the fact that these peaks were located in regions of potential regulation by transcription factors prompted us to investigate, with the web tool PhysBinder (Broos et al., 2013), the possibility that these peaks could be enriched for specific TFBS. We investigated potential hits of our ROI-DMRs to 85 TFBS motifs (all that were available in PhysBinder). For the ROI-DMR peaks of 10 kb upstream TSS and 20–40 kb downstream the TSS we combined the ROI- DMRs obtained in the BR and in the SW experiment, since the peaks were the same. For the peak at the TSS we performed independent analyses for the BR and the SW experiment since their peaks were slightly different. Interestingly, we found the following TFBS motifs with a high presence (>50%) in these ROI-DMRs: Tfcp2l1, Esrrb, RELA, and Zfx in the peak of −10 kb; Tfcp2l, Esrrb, KLF4, RELA in the peak at the TSS SW; Tfcp2l and Zfx in the peak at the TSS BR; and Tfcp2l1, Esrrb, RELA, and KLF4 in the peak of +20 to 40 kb (Supplementary Spreadsheet 3). Of particular interest is the Tfcp2l1 motif, which mapped against nearly 100% of these ROI-DMRs.

**FIGURE 7:**
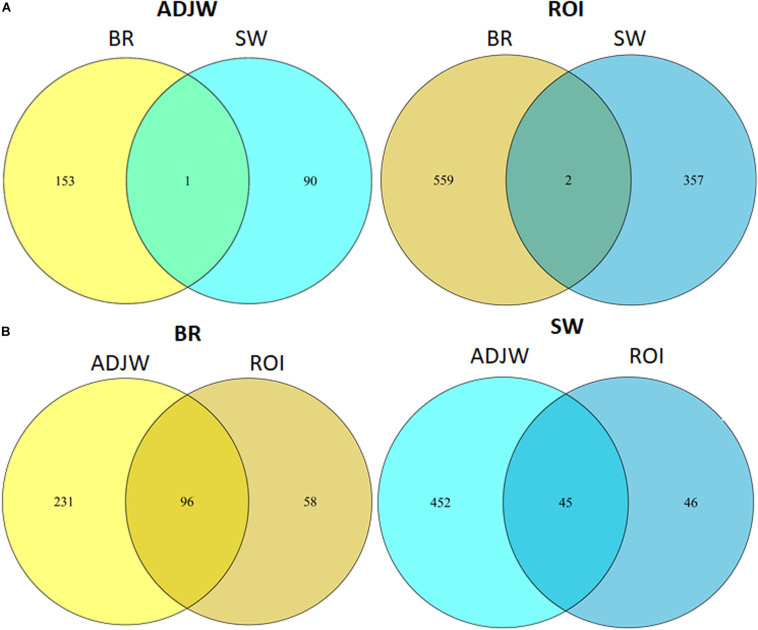
Venn diagram showing the number of ROI-DMRs overlapping between **(A)** lineages within each DMR detection method employed, and **(B)** DMR detection methods employed within each lineage.

After performing an overlap test based on permutations test (*N* = 100) between the SW and BR ROI-DMRs, we identified two common DMRs with the ADJW approach and one with the ROI approach (Figure 3). These three overlapping DMRs between BR and SW were located in Chr 2, 11, and 20. The first was located in an intronic region of a *novel gene*, the second was located in an intronic region of the *DBNDD1* gene and at the 5-prime UTR of the *CDHI* gene, while the third was in an intergenic position (Table 5). In addition, we plotted the results of the DMR overlapping test considering a maximum gap of 1 kb for each two peak ranges (Figure 7). For better visualization of DMR locations, three Manhattan plots were constructed (Supplementary Figures 2–4).

**TABLE 5:** Descriptive statistics and gene annotation DMRs found with ADJW and ROI (*P* ≤ 0.05) that overlap between BR and SW populations (S vs. C groups).

Test	Lineage	Location	Width	CpG	% of CpG	LogFC	*P*-value	FDR *p*-value	Position regarding gene	Gene symbol	ENSEMBL name	Gene strand
ADJW	BR	chr11:19110301-19110400	100	5	5%	1	1.61E-02	1.00E+00	Intron/5 prime UTR	DBNDD1/CDH1	ENSGALG00000000528/ENSGALG00000000608	−1
	SW					−2.4	4.14E-02	1.00E+00	Upstream gene	URAH/	ENSGALG00000000549/	−1
										CDH1	ENSGALG00000000608	
	BR	chr20:49501-49600	100	6	6%	−1.3	9.27E-03	1.00E+00	Intergenic	–	–	–
	SW					−4.4	4.18E-05	2.03E-01				
ROI	BR	chr2:256975-257649	675	25	4%	0.8	3.31E-02	1.00E+00	Intron	–	ENSGALG00000031276	−1
	SW	chr2:256386-257357	972	41	4%	3.5	4.35E-02	4.80E-01				

*/ DMRs commonly related to more than one gene.*

Next, we performed pathway gene enrichment analysis using ROI-DMR related genes. We identified 285 and 162 enriched pathways for BR and SW, respectively (Supplementary Spreadsheet 4). Considering an entities ratio of 0.05, which was calculated from the number of genes from a DMR-responsive gene list over the total number of genes involved in a particular canonical pathway, we found 47 pathways identifiers in common between the lineages. The pathway analysis considers the connectivity among molecules, which is represented by pathway steps. This information provides a better indication of the proportion of pathways in common between the analyzed data. All these 47 common pathways exhibit steps in common with other pathway identifiers (ratio > 0.072) (Croft et al., 2011). From these, we selected two pathways as being of special interest: cardiac conduction (FDR = 0.08) from the pathways obtained with the SW DMRs list, and Laminin interactions (FDR = 0.05) from the pathways obtained with the BR DMR list (Table 6 and Supplementary Spreadsheet 4).

**TABLE 6:** Pathways overlapping between BR and SW lineages after pathway enrichment performed with Reactome in each population using *p* < 0.05 DMRs.

Lineage	DMR location	Related gene	GENE NAME	Consequence	Pathway	
					Identifier	Name
BR	12:11592836-11593728	ENSGALG00000006802	*LAMB2*	Coding sequence, intron	R-GGA-3000157	Laminin interactions
SW	7:6634619-6634980	ENSGALG00000038311	*COL18A1*	Intron		
BR	chr1:61077944-61078198	ENSGALG00000036964	*Novel gene*	Intron	R-GGA-5576891	Cardiac conduction
SW	chr26:1304953-1305335	ENSGALG00000000329	*AHCYL1*	Coding sequence, intron		

*Functional annotation of the entities used to identify these pathways are also shown.*

## Discussion

The present study compared the RBC methylome of chickens subjected to social isolation to controls across different biomes to detect whether a common stress-related epigenetic profile (in DNA methylation) could be identified. For this, the RBC methylomic differences among lineages from different breeding programs and biomes were investigated. We performed a combined GBS + MeDIPs approach (Pértille et al., 2017a) to interrogate a reduced fraction of the RBC methylome of each individual in a pool of barcoded DNA samples. For the analysis, we used two approaches: in the first approach, a DMR call across 100 bp windows (named ADJW) was used, while in the second, pre-determined windows were used for the DMR call based on previous peak calling between the treatments (named ROI). In addition, the reduced genome of BR individuals was sequenced using the GBS approach (Pértille et al., 2016) for analysis of the genetic background.

The observation of differential methylation between treatments in specific genomic regions can sometimes be caused by confounding factors unrelated to differential immunoprecipitation (Lentini et al., 2018). To correct for this, we compared the relative differences in coverage of the inputs before immunoprecipitation. We did not identify sequencing coverage effects that interfered with the proper identification of DMRs.

Due to the massive omics data generated by NGS approaches, it is quite challenging to choose the appropriate statistic aiming to convert the data gathered into biologically relevant information. For epigenetic data, for example, commonly used software employed for obtaining DMR between treatments rely on *t*-statistics (Smyth, 2005; Luo et al., 2018), followed by statistical corrections for multiple tests. However, there is no consensus to date on which set of statistics would be the most appropriated (Korthauer et al., 2019). Many researchers have chosen to be more flexible regarding the statistical analysis, by accepting false positives but carrying out *a posteriori* analyses to integrate biological evidence into the statistics (Hoops et al., 2006; Mi et al., 2013; Croft et al., 2014; Krämer et al., 2014). In the present study, we investigated DMRs emerging from different p-value thresholds between S and C within each lineage (i.e., BR and SW). This was performed using the two above mentioned different approaches.

An interesting general parameter was that the distance among ROI-DMRs identified between the S and C groups (in both BR and SW populations) presented a multi-modal distribution with an exponential growth of the distances from 1–2 K to 20 K, 200 K, and up to 10 M. This is an unexpected pattern that deserves further investigation. We also analyzed the distance of ROI-DMRs in relation to the closest TSS and identified a trimodal distribution of the distances. Interestingly, in both populations one of the peaks of this trimodal distribution was located at the TSS, while the other two peaks were located at −10 and +20 Kbps from the TSS, (Figure 6). These distributions were consistent across chromosome types (autosomal or sexual).

The most frequent DMR peaks identified in this study were located around a gene TSS (Figure 6). This finding may have important functional consequences, since methylation levels in TSS are reported to be highly predictive of gene expression activity (Teschendorff and Relton, 2018). The peak found 10 Kbps upstream of the TSS suggests the presence of TFBS. Although studies, in general, focus on promoter-proximal regulatory regions, the influence of TF binding distant to TSS (even as far as >20 kb) to gene expression is also of relevance (McCord et al., 2011). The dynamic binding of transcription factors to TFBS is known to be affected by DNA methylation, but the specific ways this interaction occurs is still a matter of investigation (Héberlé and Bardet, 2019). Another peak identified was spread between 20 and 40 Kbps downstream of the TSS. This corresponds to terminal or downstream regions in genes. Downstream gene regions are associated with complex genetic networks in living cells, mainly at the protein regulation and degradation level (Moriya et al., 2014). The downstream boundary is also near to the downstream core promoter element (DPE), which has a particularly important role in transcription regulation by RNA polymerase II (Kadonaga, 2002).

The observation of specific peaks with a high presence of ROI-DMRs in relation to the TSS and the fact that these peaks were located in regions of potential regulation by transcription factors prompted us to investigate the possibility that these peaks could be enriched for a specific TFBS. The following TFBS motifs were highly present (>50%) in these ROI-DMRs: Tfcp2l1 and Esrrb in all ROI-DMR peaks, RELA in all but the TSS BR peak, KLF4 in the 20–40 kb downstream and TSS SW peaks, and Zfx in the 10 kb upstream and TSS BR peaks. Of particular interest is the fact that nearly 100% of these ROI-DMRs had hits against the Tfcp2l1 motif. Thus, some sequence variations of the Tfcp2l1 TFBS motif that contain CpGs could be transversal indicators of DNA methylation changes in relation to stress. Interestingly, recent research shows, in mouse and humans, that Esrrb and Klf4 are regulatory targets of Tfcp2l1 (Wang et al., 2019). Our results suggest that these effects could be mediated by DNA methylation. These are interesting findings that deserve further investigation, particularly in relation to the role of these transcription factors in the stress response.

Most of the ROI-DMRs identified in this study were located in intronic regions (36%), followed by downstream to genes (28.5%), intergenic regions (21.5%), upstream to genes (7%), and coding sequences (7%). Exon–intron boundaries have a well characterized role in guiding the splicing machinery (Schwartz and Ast, 2010). DMRs between exons, splice sites, and flanking-intronic regions are reported to be involved in the regulation of alternative splicing (Lev Maor et al., 2015). Moreover, it is known that methylation in regulatory regions (e.g., promoters) are usually associated with transcriptional repression, while in gene bodies, DNA methylation is associated with high levels of gene expression (Jones, 1999; Meissner et al., 2008; Deaton and Bird, 2011). Indeed, independent of the threshold used, most of our DMRs (including the ones overlapping between lineages) were primarily located in intronic regions, followed by down- and upstream gene regions, and coding sequences (exons) (Figure 4). Up- and downstream gene regions contain insulator elements that “protect” genes from inappropriate signals emanating from neighboring genes (Blackwood, 1998).

Differentially methylated regions were preferentially located in microchromosomes and in ChrZ in both lineages studied (Figure 5). ROI-DMRs in the sex ChrZ were present at a rate 2.38× (BR) and 4.47× (SW) higher than randomly expected. Therefore, this strongly suggests that ChrZ is a hotspot of DMRs induced by stress in RBCs. Ten ROI-DMRs were identified in the ChrZ of the BR population, while 13 ROI-DMRs were identified in the ChrZ of the SW population (*P* ≤ 0.05). Most of these DMRs were located within intronic regions of novel genes. Studies have shown an important role for sex chromosomes in the stress response (Seney et al., 2013). In humans, the sex chromosome is reported to be involved in regulating the expression of mood-related genes (Seney et al., 2013), and in mice the Y chromosome has been shown to affect several neurobehavioral traits (Sluyter et al., 1999).

In relation to specific DMRs that could be putative markers of stress, we identified 7 DMRs in the BR and 4 DMRs in the SW lineages when using a stringent threshold (*P* ≤ 0.0005) (Table 3). The only DMR that passed FDR correction (≤0.05) in both ROI and ADJW analysis was identified in the BR population. This DMR is located in Chr28 and 1,994 bps upstream the TSS of the *ADAMTSL5* gene, at its promotor region. In addition, this DMR is located downstream (≤3 kb) of the *THOP1* gene *ADAMTSL5* (thrombospondin type-1 domain-containing protein 6), is involved in the degradation of the extracellular matrix, has antiangiogenic properties, and plays an important role in inflammatory processes. Hypermethylation of the *ADAMTSL5* gene is associated with chemoresistance to acute lymphoblastic leukemia in humans (Abdullah et al., 2017). *THOP* (thimet oligopeptidase 1) is important in metabolism regulation, being broadly expressed in testis (Pineau et al., 1999). *THOP1* codes a kinase protein that uses zinc as a cofactor in humans (Gene ID: 7064). One of the mechanisms of downregulation of this gene is suggested to be hypermethylation of its promoter region (Nomoto et al., 2014). In our study, this region downstream of the *THOP1* gene was hypermethylated in the stressed group. Two significant DMRs (*P* ≤ 0.0005) were identified in ChrZ (ROI: chrZ: 7,337,093–7,337,617 and ADJW: chrZ: 70,400,401–70,400,700). One of them was in an intergenic position in SW animals and the other was located in an intron of the *UBE2R2* gene in BR animals. The *UBE2R2* gene is related to protein ubiquitination of many processes (R4GIV8_CHICK), and is reported to be one of the 14 Z-linked zebra-finch genes that has likely diverged from the Galliform lineage (Itoh et al., 2006).

We compared the DMRs obtained across our two experiments to find common DMRs emerging after social isolation stress between lineages. We were able to identify 3 DMRs (*P* ≤ 0.05, see Figure 7 and Table 5) overlapping between the BR and SW individuals. These overlapping DMRs were located on Chr2, 11, and 20 and in intronic (ENSGALG00000031276, *DBNND1* and *CDH1* genes), upstream (*URAH* and *CDH1* genes), and intergenic regions in these chromosomes, respectively. The genes associated to these DMRs are involved in cancer diseases in vertebrates. Downregulation or inactivation of the *CDH1* gene is involved in cancer progression and metastasis (Beavon, 2000). Interestingly, its regulation is dependent on many mechanisms (Acs et al., 2001) including germline mutations (Guilford et al., 1998; Pharoah et al., 2001) and promotor hypermethylation (Yoshiura et al., 1995; Stevenson et al., 2010). The *URAH* gene is associated with hepatomegaly and hepatocellular carcinoma in mice. The *ArfGAT* gene (ENSGALG00000031276) is part of the Arf family of proteins, which are involved in cancer progression, through cell–cell adhesion, integrin internalization and recycling, and actin cytoskeleton remodeling (Casalou et al., 2016). These genes can be considered strong candidates for future studies aiming at diagnosing long-term stress in the production environment by tracking epigenetic changes in RBCs of chickens.

Using subsets of gene-related DMRs (P ≤ 0.05) we performed gene enrichment analyses in SW and BR lineages. We selected two overlapping pathways found between these lineages to be discussed: “Laminin interactions” (R-HSA-3000157), which describes the action of multi-domain trimeric basement membrane proteins contributing to the structure of the extracellular matrix, thereby influencing the behavior of associated cells, such as adhesion, differentiation, migration, phenotype stability, and resistance to anoikic (Domogatskaya et al., 2012); and “cardiac conduction” (R-GGA-5576891), which involves the mechanism of the normal sequence of contraction of atria and ventricles of the heart activated by a groups of cardiac cells. The genes participating in these common pathways are mostly related to cancer and other diseases in vertebrates. Mutations in the *LAMB2* gene cause autosomal recessive Pierson syndrome, a congenital nephrotic disorder syndrome that culminates in ocular and neurologic abnormalities (Matejas et al., 2010). The *COL18A1* gene is associated with neovascularization and vascular permeability in atherosclerosis in mice (Moulton et al., 2004) and is also reported to inhibit angiogenesis and tumor growth (Arvanitidis and Karsdal, 2016). The *AHCYL1* gene is a estrogen-stimulated gene expressed in the chicken oviduct affecting growth, development, and calcium metabolism of the mature oviduct of hens (Jeong et al., 2012b). *AHCYL1* expression is associated with ovarian carcinogenesis as an oncogene in chickens, however, has a paradoxical effect as a tumor suppressor in human epithelial ovarian cancer (Jeong et al., 2012a).

These findings are relevant for the future identification of epigenetic markers of stress in RBCs, a tissue that is subjected to systemic hormonal fluctuations caused by a centrally produced stress response. Curiously, all levels of analyses performed in this study showed that basically all genes related to stress-responsive DMRs in RBCs (caused by the environmental effect) have already been studied and related to abnormalities or disorders. These abnormalities are often considered to be inherited or represent idiopathic etiologies but are not generally associated with epigenetic alterations caused by detrimental exposures such as long-term stress.

## Conclusion

This paper describes potential candidate genes for stress diagnosis across layer populations of chickens reared in different biomes. With a *P*≤0.05 we found 4 genes related to 3 DMRs observed in both populations analyzed here. Among the gene-related DMRs obtained, we also found 4 genes related to the top two biological pathways enrichedin both BR and SW populations. These DMRs were within or nearby (3 ≤ kb far) genes. In addition, we provide a robust list of DMRs related to an experimental condition that is well known to generate stress response and consequently, compromise the animals’ health. Interestingly, the sexual ChrZ had a DMR rate higher than randomly expected, indicating that this chromosome is a strong candidate for screening for epigenetic changes caused by stress. The biological functions of the genes associated to the DMRs found in our study, and their related enriched pathways, are relevant for the function of the tissue investigated (RBC). Our results suggest that most of the identified DMRs are enriched near TSS, DPE, and specific TFBS. These DMRs could be tested more extensively as markers for long-term stress diagnosis in order to monitor good practices in real life animal production setups.

## Data Availability Statement

The datasets generated for this study can be found in the ENA, http://www.ebi.ac.uk/ena/data/view/PRJEB34868.

## Ethics Statement

All animal experimental protocols employed in the present study were performed in accordance with international guidelines for animal welfare. In Brazil, the study was approved by the resolution #008/2017 from the Embrapa Swine and Poultry Ethics Committee on Animal Utilization (CEUA) following the National Council for the Control of Animal Experimentation (CONCEA). In Sweden the study was under the license #50-13 from the Regional Committee for Scientific Research on Animals from Linköping, Sweden.

## Author Contributions

FP analyzed the data and wrote the manuscript with the valuable help of CG-B and MP. PJ and CG-B were responsible for the experiments design in Sweden, while LC, ML, and FP were responsible for the experiments design at the Brazilian facilities. AI and ML were responsible for conducting the field experiments carried out in Brazil, while MS conducted the experiments in the Swedish facilities, which included tissue sampling and DNA extraction. MS, AF, SR, and AI carried out the lab work for sequencing library prep. LC, ML, PJ, and CG-B provided the lab and office infrastructure. LC, ML, CG-B, and PJ provided overall supervision for the research activities. The final version of the manuscript was produced by FP and CG-B. All authors reviewed the final drafts and approved the last version of the manuscript.

## Conflict of Interest

The authors declare that the research was conducted in the absence of any commercial or financial relationships that could be construed as a potential conflict of interest.

## Footnotes

1 https://www.bioconductor.org/packages/release/bioc/vignettes/MEDIPS/inst/doc/MEDIPS.pdf

2 https://github.com/taoliu/MACS/

3 http://bioit.dmbr.ugent.be/physbinder/index.php

4 http://bioit.dmbr.ugent.be/physbinder/models.php

5 www.reactome.org

## Abbreviations

ADAMTSL5: thrombospondin type-1 domain-containing protein 6
ADJW: adjacent windows
BR: Brazil
SW: Sweden
C: control animals
CEUA: Ethics Committee on Animal Utilization
CNPq: National Council for Scientific and Technological Development
CONCEA: National Council for the Control of Animal Experimentation
CP: crude protein
DMRs: differentially methylated regions
EMBRAPA: Brazilian Agricultural Research Corporation
FAPESP: S o Paulo Research Foundation
FORMAS: Swedish Research Council for Sustainable Development
GBS: genotyping by sequencing
HPA: hypothalamic-pituitary-adrenal
IGF2R: insulin-like growth factor 2
Iset1: input set 1
Iset2: input set 2
MACS2: model-based analysis of ChIP-Seq data version 2
ME: metabolizable energy
MeDIP: methylated DNA immunoprecipitation
minRowSum: minimum sum of counts
mnMAF: minimum minor allele frequency
mnScov: minimum site coverage
mnTCov: minimum taxon/sample coverage
RBC: red blood cells
ROI: regions of interest
S: social isolation treatment
SP: S o Paulo
TFBS: transcription factor binding site
THOP: thimet oligopeptidase 1
TMM: trimmed mean of M values
VEP: variant effect predictor.
