# Supplementary figures and images for: Putative Epigenetic Biomarkers of Stress in Red Blood Cells of Chickens Reared Across Different Biomes
Source: Front Genet. 2020 Nov 2;11:508809. doi: 10.3389/fgene.2020.508809 (PMC7667380; doi:10.3389/fgene.2020.508809)

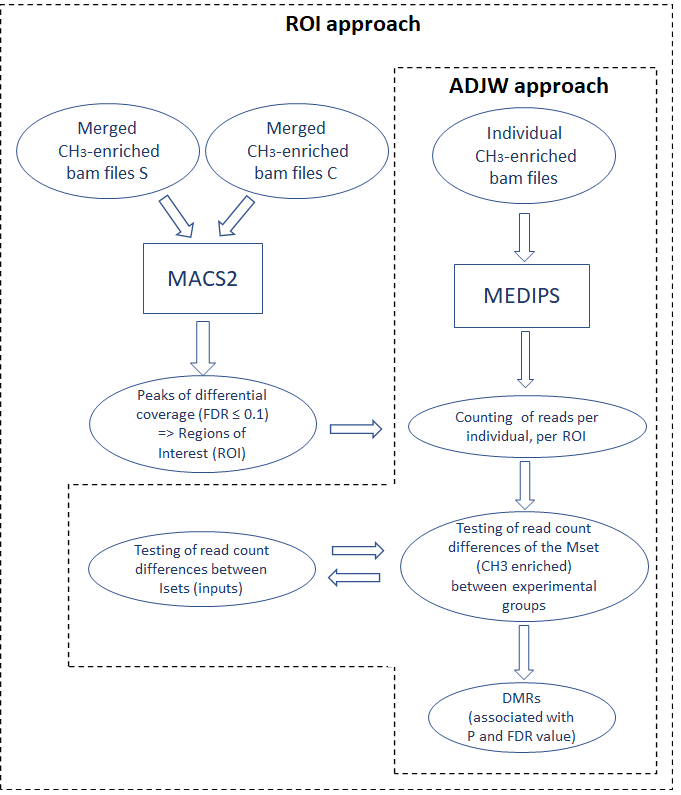

Supplement: Supplementary file 1 [file Image_1.TIF]
