# Supplementary material for: Putative Epigenetic Biomarkers of Stress in Red Blood Cells of Chickens Reared Across Different Biomes
Source: Front Genet. 2020 Nov 2;11:508809. doi: 10.3389/fgene.2020.508809 (PMC7667380; doi:10.3389/fgene.2020.508809)
Supplement: Supplementary file 9 [file Presentation_1.pdf]

Supplementary Table S1. Sequencing and alignment statistics for the Brazilian White Leghorn lineage

Samples	Depth	bp Sequenced	Breadth (bp Sequenced/depth)	% of the *Gal.gal* genome v.5.0
7015_MeD1	31.7	295408723	9312189.4	0.87
7016_MeD2	25.6	832172066	32458414.1	3.02
7020_MeD1	25.2	189546154	7530578.0	0.70
7021_MeD2	27.8	1003311203	36121385.0	3.36
7022_MeD1	33.8	1225330777	36247027.9	3.37
7023_MeD2	25.4	192182531	7554879.3	0.70
7027_MeD1	32.6	1304734864	39971045.4	3.72
7028_MeD2	29.6	393804620	13301603.7	1.24
7032_MeD1	28.8	984802938	34171765.3	3.18
7033_MeD2	30.5	459302304	15078966.5	1.40
7034_MeD1	25.0	720493706	28793488.6	2.68
7035_MeD2	29.0	441295599	15194193.5	1.41
7037_MeD1	34.1	479456188	14069747.4	1.31
7038_MeD2	28.5	575777754	20228777.2	1.88
7041_MeD1	35.3	445276355	12625971.7	1.17
7042_MeD2	31.2	470414147	15060771.0	1.40
7046_MeD1	31.6	1191563338	37753937.2	3.51
7047_MeD2	29.6	433131327	14610307.3	1.36
7053_MeD1	33.9	462793117	13641975.8	1.27
7054_MeD2	26.3	780340135	29679642.7	2.76
7055_MeD1	29.2	368092791	12605097.3	1.17
7056_MeD2	31.6	384803020	12183633.9	1.13
7058_MeD1	23.1	634030676	27451969.0	2.55
7059_MeD2	27.9	1024884425	36795402.6	3.42
7060_MeD1	33.1	427452168	12922669.0	1.20
7061_MeD2	32.6	430368078	13182307.9	1.23
7069_MeD1	31.6	526618575	16650075.4	1.55
7070_MeD2	34.1	493849470	14489867.8	1.35
7078_MeD1	30.8	491468744	15978150.8	1.49
7079_MeD2	32.3	553372862	17120148.9	1.59
7080_MeD1	31.7	462173602	14600334.9	1.36
7081_MeD2	32.4	436780887	13474029.8	1.25
7015_input1	24.3	768825540	31633053.1	2.94
7016_input2	26.7	928586824	34751070.3	3.23
7020_input1	24.5	824291960	33598902.7	3.13
7021_input2	22.0	777816462	35301378.9	3.28
7022_input1	26.4	914468705	34669696.1	3.23
7023_input2	25.2	844495123	33468812.7	3.11
7027_input1	23.6	666364228	28274590.0	2.63

7028_input2	23.1	724726481	31338032.8	2.92
7032_input1	19.0	535820492	28134148.9	2.62
7033_input2	18.3	554995745	30405227.8	2.83
7034_input1	19.5	581623116	29859442.2	2.78
7035_input2	22.9	694511191	30283302.0	2.82
7037_input1	22.0	643137379	29246412.2	2.72
7038_input2	32.2	1279677373	39686933.0	3.69
7041_input1	22.4	680844300	30403793.1	2.83
7042_input2	20.8	659574765	31657056.2	2.94
7046_input1	20.7	691598186	33434930.1	3.11
7047_input2	22.1	760937592	34360822.4	3.20
7053_input1	19.3	488638882	25365653.8	2.36
7054_input2	20.4	587823560	28878441.3	2.69
7055_input1	19.2	535161417	27916464.5	2.60
7056_input2	21.1	715608621	33993721.1	3.16
7058_input1	33.7	1389053102	41215380.0	3.83
7059_input2	22.5	701101585	31178223.1	2.90
7060_input1	21.5	726590471	33771344.2	3.14
7061_input2	23.3	678911047	29189050.6	2.72
7069_input1	23.1	743286547	32217771.8	3.00
7070_input2	23.1	739180764	32011362.1	2.98
7078_input1	20.8	686583876	33069573.7	3.08
7079_input2	21.7	656157576	30249106.9	2.81
7080_input1	26.5	815248418	30744136.6	2.86
7081_input2	22.9	733997562	31999893.7	2.98
**TotalAverage**	25.8	662218637.7	26479622.2	2.46
**TotalSD**	5.2	248211623	9150209	0.85
**MeDIPAverage**	30.2	597344785.8	20026886.1	1.86
**MeDIPSD**	3.1	294204155.2	10085067.7	0.94
**InputAverage**	23.0	741551215	31947116.0	2.97
**InputSD**	3.4	186608136	3230589.0	0.30

*MeD means that individuals were subjected to GBS+MeDIP approach, and input means that individuals were subjected to GBS approach only. The numbers 1 and 2 represent control and isolated groups, respectively.

Supplementary Table S2. Sequencing and alignment statistics for the Swedish White Leghorn lineage

Samples	Depth	bp Sequenced	Breadth (bp Sequenced/depth)	% of the *Gal.gal* genome v.5.0
9103_MeD2	58.6574	217291466	3704416.9	0.34
9104_MeD2	56.8586	228587592	4020281.8	0.37
9105_MeD2	66.9302	291254510	4351615.7	0.40
9109_MeD2	51.5768	386200724	7487876.8	0.70
9116_MeD2	45.8561	197302675	4302648.4	0.40
9117_MeD2	54.0269	438148433	8109820.0	0.75
9118_MeD2	52.6391	192624836	3659349.0	0.34
9120_MeD2	53.9478	260009946	4819658.0	0.45
9130_MeD1	58.4201	270670524	4633174.6	0.43
9132_MeD1	49.5792	282396940	5695875.3	0.53
9134_MeD1	50.4113	626865258	12435014.7	1.16
9136_MeD1	53.3895	207347664	3883678.7	0.36
9148_MeD1	56.6508	423679222	7478786.2	0.70
9154_MeD1	58.2284	326872583	5613628.1	0.52
**MEDIPAverage**	54.8	310660883.8	5728273.2	0.5
**MEDIP SD**	5.1	121917627.9	2441239.6	0.2

*MeD means that individuals were subjected to GBS+MeDIP approach. The numbers 1 and 2 represent control and isolated groups, respectively

Supplementary Table S3. Comparison between the enrichment of CpG in genomic regions covered by the set of sequenced *reads* (seq - White Leghorn line of Brazil) and reference genome (genome)

	seq / genome (nº of CpGs)		seq / genome (relH)		seq / genome (GoGe)		enrichment.score (relH)	enrichment.score (GoGe)	% of CpGs from *Gal.gal* genome v.6.0
**Media Total**	443891		1.78		0.28		1.59	1.07	**3.9**
Media (Inp1)	528605		1.68		0.27		1.50	1.04	**4.6**
Media (Inp2)	541815	/11403014	1.67	/1.116	0.27	/0.262	1.50	1.04	**4.8**
Media (Inp3)	510815		1.69		0.27		1.51	1.04	**4.5**
Media (MeD1)	346586		1.89		0.29		1.69	1.11	**3.0**
Media (MeD2)	320912		1.92		0.29		1.72	1.12	**2.8**

Legend: *relH* and *GoGe* show the relative frequency and the observed/expected CpG relation; enrichment.score.relH and enrichment.score.GoGe indicate the enrichment of CpGs within the regions sequenced in each experiment compared to a non-enriched reference genome, respectively

Supplementary Table S4. Comparison between the enrichment of CpG in genomic regions covered by the set of sequenced *reads* (seq - White Leghorn line of Sweden) and reference genome (genome)

	seq / genome (nº of CpGs)		seq / genome (relH)		seq / genome (GoGe)		enrichment.score (relH)	enrichment.score (GoGe)	% of CpGs from *Gal.gal* genome v.6.0
**Media Total**	132206		3.21		0.402		2.87	1.53	**1.2**
Media (MeD2)	112683	/11403014	3.24	/1.116	0.404	/0.262	2.90	1.54	**1.0**
Media (MeD1)	154997		3.16		0.399		2.83	1.52	**1.4**

Legend: *relH* and *GoGe* show the relative frequency and the observed/expected CpG relation; enrichment.score.relH and enrichment.score.GoGe indicate the enrichment of CpGs within the regions sequenced in each experiment compared to a non-enriched reference genome, respectively
